# Active directional switching of surface plasmon polaritons using a phase transition material

**DOI:** 10.1038/srep43723

**Published:** 2017-03-06

**Authors:** Sun-Je Kim, Hansik Yun, Kyungsoo Park, Jongwoo Hong, Jeong-Geun Yun, Kyookeun Lee, Joonsoo Kim, Sun Jae Jeong, Sang-Eun Mun, Jangwoon Sung, Yong Wook Lee, Byoungho Lee

**Affiliations:** 1 Inter-University Semiconductor Research Center and School of Electrical and Computer Engineering, Seoul National University, Gwanak-Gu Gwanakro 1, Seoul, 08826, Korea; 2 Interdisciplinary Program of Biomedical Mechanical & Electrical Engineering and School of Electrical Engineering, Pukyong National University, Yongso-ro 45, Nam-Gu, Busan, 48513, South Korea

## Abstract

Active switching of near-field directivity, which is an essential functionality for compact integrated photonics and small optoelectronic elements, has been challenging due to small modulation depth and complicated fabrication methods for devices including active optical materials. Here, we theoretically and experimentally realize a nanoscale active directional switching of surface plasmon polaritons (SPPs) using a phase transition material for the first time. The SPP switching device with noticeable distinction is demonstrated based on the phase transition of vanadium dioxide (VO_2_) at the telecom wavelength. As the insulator-to-metal phase transition (IMT) of VO_2_ induces the large change of VO_2_ permittivity at telecom wavelengths, the plasmonic response of a nanoantenna made of VO_2_ can be largely tuned by external thermal stimuli. The VO_2_-insulator-metal (VIM) nanoantenna and its periodic array, the VIM metagrating, are suggested as optical switches. The directional power distinction ratio is designed to change from 8.13:1 to 1:10.56 by the IMT and it is experimentally verified that the ratio changes from 3.725:1 to 1:3.132 as the VIM metagratings are heated up to 90 °C. With an electro-thermally controllable configuration and an optimized resonant design, we expect potential applications of the active switching mechanism for integrable active plasmonic elements and reconfigurable imaging.

For the last few decades, much attention has been paid to optical technologies as integration density and signal delay have been the bottlenecks in the improvement of integrated electronic systems^1^,^2^,^3^. Optical components can offer a significantly high operating frequency with large bandwidth and low power dissipation^1^,^2^,^4^. However, the considerable size mismatch has been the obstacle of interconnecting optical circuit elements and conventional integrated electronic circuits. As a promising candidate for scaling down optical components, surface plasmon polaritons (SPPs) have attracted enormous interest owing to the capability of overcoming the diffraction limit of light^5^,^6^,^7^,^8^,^9^. Numerous plasmonic nanostructures guiding and modulating SPPs have been suggested for integrable ultracompact optical components. In the field of active plasmonics, many researchers have demonstrated tunable plasmonic modulators exploiting active optical materials and electro-optic nonlinear effects to develop integrable active plasmonic components^10^,^11^,^12^,^13^,^14^,^15^,^16^,^17^. Particularly, there have been lots of efforts on directive emission of SPPs^18^,^19^,^20^,^21^,^22^,^23^,^24^,^25^ and optical beams^26^,^27^,^28^ by using plasmonic nanoantennas for efficient launching or focusing of SPPs. Active modulation of SPP directivity is an essential functionality for building compact optoelectronic system and reducing size of integrated photonic system. However, as the directivity modulation mechanisms of these devices depend on external control of incident beams, including polarization state, phase difference of multiple beams, and incident angles, a bulky configuration with multiple optic components is inevitable^18^,^19^,^21^,^22^,^23^,^24^,^25^. Hence, demonstration of SPP directivity switching using active optical materials for nanoscale near-field modulation would be highly desirable and useful.

There are several candidates of active optical materials for nanoscale modulation of SPP signals. Indium tin oxide, a transparent conducting oxide material exhibiting plasmonic property in telecom wavelengths, has been widely utilized for field-effect plasmonic modulators^10^,^11^,^16^. Graphene, another rising candidate for electro-optically controllable plasmonic material, has been mainly utilized for active plasmonic waveguide modulators. However, devices based on these materials suffer from low modulation depth and low operating frequencies, respectively, so that they are not appropriate to be utilized for nanoscale directional switching actions of light in optical frequencies.

On the other hand, the insulator-to-metal transition (IMT), which is observed in vanadium dioxide (VO_2_), gives a promising route for active switching in the visible and infrared frequencies^29^,^30^,^31^. When VO_2_ is heated over the critical temperature about 68 °C, a VO_2_ thin film undergoes the IMT due to structural transformation from monoclinic to rutile lattice. Drastic changes in material properties follow the IMT in terms of electronic conductivity and optical permittivity. It has been reported that the real part of the VO_2_ permittivity varies from positive to negative value in optical frequencies as the VO_2_ phase changes from the insulator phase to the metal phase^30^,^31^. A large permittivity change of VO_2_ has attracted many research groups. Novel VO_2_-based plasmonic modulators for waveguide modes, tunable hot spot, and far-field scattering spectra controlled by external thermal, electrical, and optical stimuli have been suggested^17^,^31^,^32^,^33^,^34^,^35^,^36^,^37^,^38^,^39^. However, active directional switching of asymmetric SPP excitation using a phase transition material has not been reported yet to the best of our knowledge.

In this paper, we propose a novel reversible thermo-optical SPP switch that can change the direction of the asymmetrically excited SPPs. Power directivity of SPPs can be switched as VO_2_ slabs comprising nanoantennas undergo the phase transition induced by external heating. Computational and experimental results show that power distinction ratio of generated SPPs is switched according to the device temperature. The proposed structure allows SPPs to switch their direction without any modulation of incident light. We firstly analyze the SPP excitation characteristics of a symmetric VO_2_–insulator–metal (VIM) nanoantenna, which is a composite structure of VO_2_–SiO_2_–Au slabs, for the insulator phase and the metal phase, respectively. Then, we design an asymmetric VIM composite nanoantenna with an additional nanoslit in it (Fig. 1(a)) in order to tune the directional launching of SPPs on the backside boundary between the gold layer and the sapphire substrate. Furthermore, we propose and experimentally demonstrate a tunable metagrating (Fig. 1(b)) which is an array of the above-mentioned VIM nanoantennas having the period of backside SPP wavelength, for the sake of efficient SPP launching with high intensity.

## Device Concepts and Design Principles

**Figure 2:**
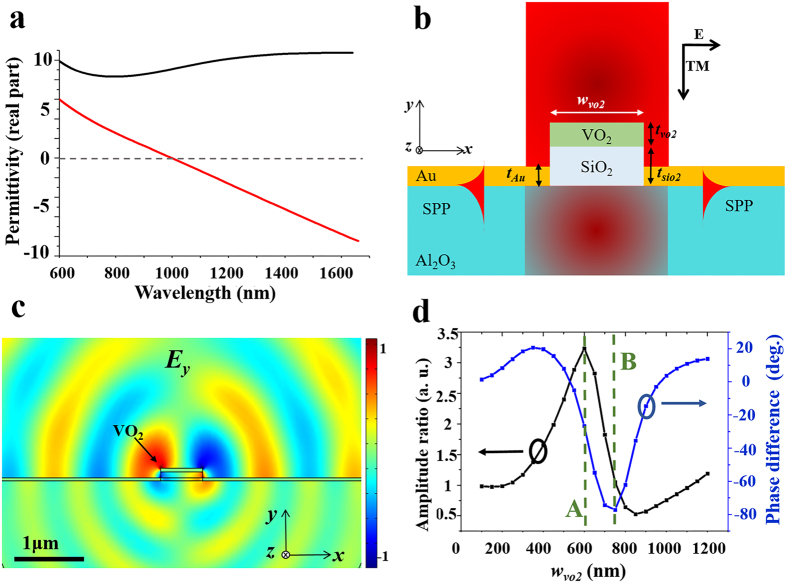
(**a**) Measured near-infrared permittivity (real part) spectra of the VO_2_ film at room temperature (the upper black curve) and 90 °C (the lower red curve). (**b**) A scheme of the symmetric VIM nanoantenna launching SPPs (*t*_*Au*_ = 50 nm, *t*_*sio2*_ = 150 nm, *t*_*vo2*_ = 50 nm). (**c**) Ey-field profile of the wide symmetric VIM nanoantenna in the insulator phase under incident light of 1650 nm wavelength. (**d**) IMT-induced amplitude ratio and phase difference between backside SPPs launched in insulator and metal phases according to *w*_*vo2*_.

The permittivities of VO_2_ in both insulator and metal phases are very sensitive to the deposition method or process of the VO_2_ film in general. Hence, ellipsometry measurements on a VO_2_ film both below and above the phase transition temperature (~68 °C) are the essential step for the design of VO_2_-included nanostructures. Considering high optical loss of VO_2_, the deposition thickness is chosen to be 50 nm. The measured spectra of real part permittivities in both insulator and metal phases in near-infrared wavelengths are shown in Fig. 2(a). Around the telecom wavelength, the real part of the VO_2_ permittivity changes its sign as the phase transition occurs and the difference of the permittivities for two phases is fairly large. To effectively utilize large variation of induced dipole moments on the VO_2_ slab, a working wavelength is set to be 1650 nm. Using the measured optical properties, the SPP launching characteristics of the VIM nanoantenna are analyzed according to the VO_2_ phase. A SiO_2_ layer of 150 nm thickness is deposited between the 50 nm-thick gold film and the 50 nm-thick VO_2_ slab to avoid direct contact between metallic VO_2_ and gold. If the VO_2_ slab is inserted within the gold film, coupling efficiency of the VIM nanoantenna is reduced significantly when VO_2_ is in the metal phase. The thickness of the SiO_2_ slab is chosen not to be too thick so that scattering properties of launched backside SPPs are highly affected by the phase of the VO_2_ slab, which is located slightly above the gold film.

When the VIM nanoantenna is normally illuminated by a monochromatic laser with TM-polarization as depicted in Fig. 2(b), two SPP modes with different wavelengths are symmetrically excited on opposite sides of the gold film which is shown in Fig. 2(c). The backside SPP mode is investigated between the two modes in order to separate output signals from the input beam more clearly. IMT-induced variations of backside SPPs in terms of optical phase and amplitude are highly dependent on the width of the VIM nanoantenna as described in Fig. 2(d). In particular, a large amount of variation occurs at two values of antenna width (*w*_*vo2*_), 600 nm and 750 nm, which are marked by dashed lines denoted as A and B in Fig. 2(d), respectively. When the VIM width is about 750 nm, phase of the backside SPPs changes by nearly 80° while the amplitude does not change significantly owing to the IMT. It implies that the nanoantenna is suitable for directional switching of SPPs. On the other hand, when *w*_*vo2*_ is about 600 nm, the power of the launched SPPs in each phase differs by roughly 10.6 times. Hence, this condition can be useful for on-off switch of backside SPP launching rather than transmissive directional SPP switching. That is, the VIM antenna with the width of around 750 nm is adequate for directional switching of SPPs.

**Figure 3:**
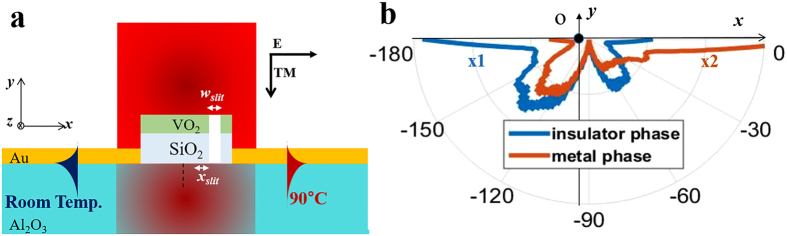
(**a**) A Scheme of the asymmetric VIM nanoantenna for directional switching. (**b**) Angular distribution of propagating power carried by backside near-fields in the both insulator and metal phases. *w*_*vo2*_, *w*_*slit*_, and *x*_*slit*_ are set to be 750 nm, 150 nm, and 100 nm, respectively.

Based on the analysis of the symmetric case in the previous section, the VIM nanoantenna for asymmetric SPP launching is designed. The schematic illustration of the structure is described in Fig. 3(a). A nanoslit in the VIM nanoantenna breaks the symmetry of the structure so that angular distributions of backside SPP scattering patterns show asymmetric directivities. As the 0th-order far-field transmission is dominant in case of the symmetric VIM (see part 1 of Supplementary Information), asymmetric insertion of the nanoslit into the symmetric VIM can be understood as a small perturbation in the spatial permittivity profile of the VIM structure^40^. Hence, it can significantly change the scattering patterns of the backside SPPs while the 0th-order transmission, the main stream of the forward scattering, is not perturbed that much. Width (*w*_*slit*_) and position (*x*_*slit*_) of the nanoslits are optimized by parametric studies where the focused ion beam (FIB) milling resolution is taken into account with *w*_*vo2*_ fixed as 750 nm. In Fig. 3(b), angular spectra of the excited backside SPPs in the insulator and metal phases are depicted when the antenna is optimized by parametric studies. The optimal values of *w*_*slit*_ and *x*_*slit*_ are 150 nm and 100 nm, respectively, which make distinction ratios of both phases similar (see part 2 of Supplementary Information). In the insulator phase, the power of the left-directed SPPs is 3.8 times larger than that of the right-directed ones. On the other hand, in the metal phase, the power of the right-directed SPPs is 8.81 times higher than that of the left-directed ones. It is also shown that the forward scattering is not much affected by the IMT of the VIM nanoantenna while backside SPPs are switched as expected (see part 1 of Supplementary Information).

**Figure 4:**
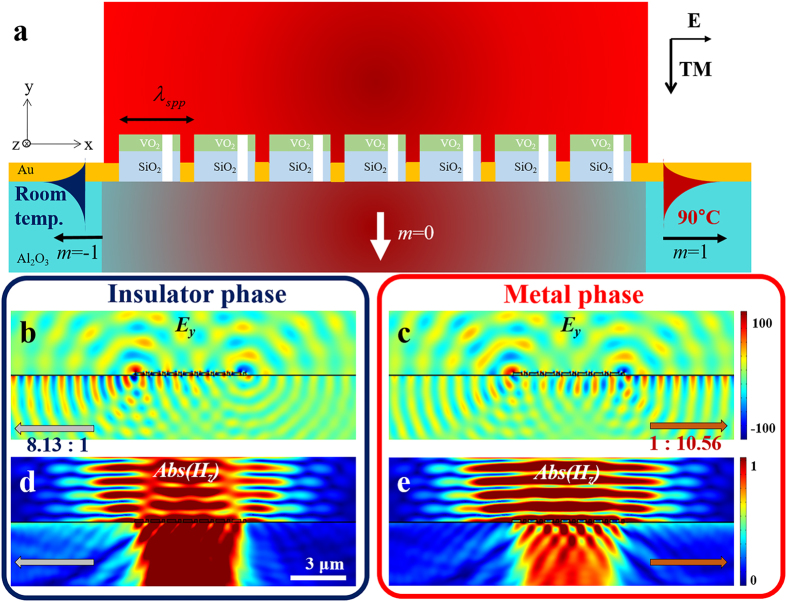
(**a**) Scheme of the VIM metagrating in the metal phase. The proposed VIM metagrating is formed by periodic arrangement of 7 asymmetric VIM nanoantennas with a period of 932 nm. *E*_*y*_-field profiles in (**b**) insulator and (**c**) metal phases, respectively. Magnitude profiles of transverse magnetic fields in (**d**) insulator and (**e**) metal phases, respectively. Tightly focused Gaussian beams with a waist of 5 μm are imposed in common for the calculations of (**b**–**e**). Left to right power distinction ratio of backside SPPs in the insulator phase, as depicted graphically in (**b**,**d**), is about 8.13. Right to left power distinction ratio of backside SPPs in the metal phase, as depicted graphically in (**c**,**e**), is about 10.56.

Although a single asymmetric VIM nanoantenna can be utilized as a directional SPP coupler with thermo-optical switching functionality, SPP coupling efficiency of the single VIM nanoantenna, which is about 0.24%, is too low to be integrated into plasmonic systems and other applications. Moreover, it is not easy to verify the performance of the nanoantenna experimentally due to its low efficiency. Hence, we propose a more efficient directional SPP switch which we named as a VIM metagrating. As shown in Fig. 4(a), the proposed structure is a periodic array of asymmetric VIM nanoantennas with a period of 932 nm, which is equal to the wavelength of backside SPPs. Each VIM nanoantenna comprising the metagrating exhibits directional SPP launching characteristics in the insulator and metal phases.


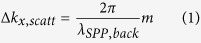


Backside SPPs are basically launched by an additional momentum in the x-direction given by the period of the VIM metagrating, as described in Eq. 1. The positive first-order diffraction, denoted as *m* = 1 in Fig. 4(a), implies right-directed backside SPPs. The negative first-order diffraction, *m* = −1 in Fig. 4(a), implies left-directed backside SPPs. As a single asymmetric VIM antenna is designed to launch backside SPPs with the distinguished directivities in the insulator and the metal phases, the two first order diffractions, *m* = 1 and *m* = −1, by the VIM metgrating are suppressed in the insulator phase and the metal phase, respectively. Hence, the whole metagrating excites the 0th-order transmission and directional SPPs with opposite directivities in both insulator and metal phases. If directional distinction ratios and switching property of SPP power are preserved in similar levels compared to those of the single asymmetric VIM nanoantenna, higher SPP coupling efficiency can be achieved owing to larger geometric cross-section of the device compared to the single VIM nanoantenna. When *w*_*vo2*_, *w*_*slit*_, *x*_*slit*_, the period, and the number of the periodic VIM nanoantennas are 750 nm, 150 nm, 100 nm, 932 nm, and 7, the ratios of directionally launched SPP power are 8.13:1 and 1:10.56 in the insulator and metal phases, respectively. Figure 4(b–e), *Ey* and Abs(*H*_*z*_) profiles in both phases, clearly show directional SPP launching characteristics with opposite directivities in both phases.

## Experimental Results

**Figure 5:**
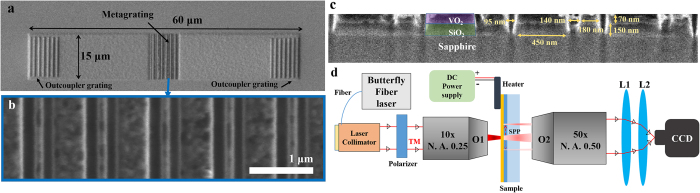
(**a**,**b**) SEM images of the final device including the VIM metagrating and two outcoupler gratings, and (**c**) its cross-section. (**d**) Experimental apparatus of optical microscopy setup for SPP signal detection in far-fields.

**Figure 6:**
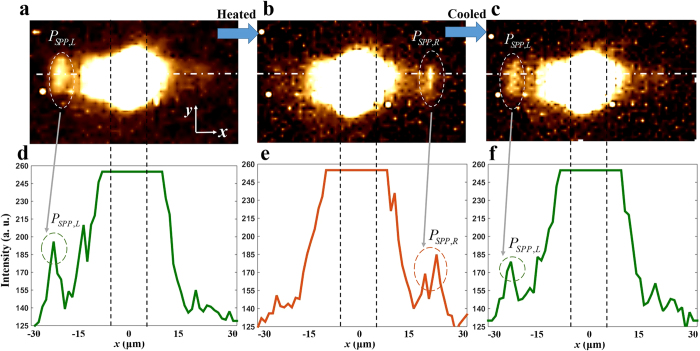
IR CCD images when the sample is (**a**) in room temperature, (**b**) heated up to 90 °C, and (**c**) cooled down to room temperature. (**d**–**f**) Cross-sectional data of far-field intensities according to *x*-coordinate extracted from (**a**–**c**) respectively. The lines where intensity data are extracted are depicted in (**a**–**c**) as white dash-dotted lines. The black dashed lines in (**a**–**c**) indicate position of the sample along the *x*-axis.

The designed VIM metagrating SPP switch is fabricated through 5 steps; three material deposition steps and two FIB milling steps (see part 3 of Supplementary Information). As a result, in the middle of two regions with two outcoupler gratings, the VIM metagrating is formed with periodic arrangements of 7 asymmetric VIM nanoantennas with a period of 930 nm. SEM images of the final device are shown in Fig. 5(a–c). To verify directional launching and switching of SPPs experimentally, we utilize a microscopy setup using two outcoupler gratings^22^,^41^ (Fig. 5(d). A butterfly fiber laser with the wavelength of 1650 nm (Thorlabs, FPL1059S) is collimated and focused on the metagrating sample with a tight beam waist below 20 μm by using an objective lens. When a focused beam illuminates the sample, transmitted backside signals are gathered by another objective lens and imaged by two lenses and an NIR CCD. Firstly, we measure CCD images at room temperature. Then, the sample is heated over the phase transition temperature, 90 °C in this case, by using an attached electronic heater and a DC power supply. Finally, the sample is cooled down again to room temperature, and CCD images are measured again to check switching reversibility. The temperature of the sample is monitored by using an IR laser thermometer (Testo, 830-T1). When you see Fig. 6(a and b), the measured NIR CCD images in the insulator phase and the metal phase, clear bright spots at the positions where outcoupler gratings are located are seen at the right and left sides of the metagrating sample, respectively. It directly refers that the IMT of VO_2_ induces the directivity switching of asymmetrically launched backside SPPs. Also, when the sample is cooled from 90 °C to the room temperature, as shown in Fig. 6(c), position of bright outcoupled signal is reversed back to the position of the left outcoupler grating. Hence, it is verified that the directional launching and switching of SPPs are reversible according to the phase of VO_2_ retained in the metagrating. For quantitative analysis of the device performance, we plotted three cross-sectional intensities of transmitted and outcoupled SPP signals along the x-direction, Fig. 6(d–f). The distinction ratios of overall integrated SPP energies outcoupled from the left and the right gratings are 3.725:1, 1:3.132, and 2.713:1 in those cross-sectional intensity plots, Fig. 6(d–f). Distinction ratios of SPP powers are calculated considering common intensity offset level, 125, in Fig. 6(d–f).

## Conclusion

In conclusion, we theoretically and experimentally demonstrate a reversible active switching of SPP directivity via thermally induced IMT of VO_2_. At first, the VIM nanoantenna is designed as a SPP switching element and then its periodic array, the VIM metagrating, is optimized for higher intensities of coupled SPP signals. In the final step, experimental demonstration of the VIM metagrating verifies that the IMT of a deep subwavelength-sized VO_2_-included nanoantenna is able to switch scattering directions of optical near-fields depending on the temperature. Moreover, it is verified that the switching action is reversible depending on the temperature of the sample. The efficiency of directional SPP launching and switching can be improved if the proposed mechanism is applied to optically resonant nanoantennas or reflection-type configurations rather than transmission type, with more elaborate lithography techniques. Moreover, by utilizing a resonant and reflective configuration, the number of VIM nanoantenna composing the VIM metagrating can be reduced for smaller footprint of the device. Our demonstration and novel idea would be meaningful as a proof-of-concept not only to researchers studying plasmonics but also to people developing various active devices for optical beam steering. The proposed novel directional switching mechanism using a phase transition material can be well integrated with conventional CMOS electronic circuits if the IMT of VO_2_ is controlled in electro-thermal manner within nanoscale. Furthermore, it also promises potential applications including integrated photonic systems, ultracompact thermo-optical sensors, near-field heat transporting devices, and various reconfigurable imaging systems.

## Methods

### Sample fabrication

The device was fabricated by three deposition methods and the FIB millings. First, a 150 nm-thick SiO_2_ layer was deposited on a c-plane sapphire (Al_2_O_3_) wafer by plasma enhanced chemical vapor deposition (Oxford instruments, PlasmaPro System 100). We used pulsed laser deposition (LAMBDA PHYSIK, COMPEX 205) with a KrF excimer laser at 248 nm^42^ for deposition of a 50 nm-thick VO_2_ film on the SiO_2_ layer. In the third step, two large background regions (45 μm by 15 μm) were formed using an FIB milling machine (FEI, Quanta 200 3D) by removing VO_2_ and SiO_2_ layers except for the VIM metagrating. Then, the periodic arrangement of the 7 asymmetric VIM nanoantennas was milled between those two background regions by utilizing high-resolution milling. Then, the 50 nm-thick gold film was deposited on the VO_2_ layer by using an e-beam evaporator (MUHAN, MHS-1800). In the last step, using the FIB machine again, two outcoupler gratings were formed 30 μm far from the metagrating to convert propagating backside SPPs into normally scattered far-fields on both left and right sides of the device. Also, the gold residues deposited on the VO_2_ slabs and inside the nanoslits were raked out.(see part 3 of Supporting Information)

### Material properties

Optical properties of the 50 nm-thick VO_2_ film were measured by using a variable angle spectroscopic ellipsometer system (J. A. Woollam) in the insulator and metal phases. The measurements in each phase were made at room temperature and at 90 °C, which is high enough to induce homogeneous and stable IMT of the sample, respectively. We used the permittivity data of gold and silicon dioxide measured by Palik, E.D^43^. and Malitson, I. H^44^.

## Additional Information

**How to cite this article:** Kim, S.-J. *et al*. Active directional switching of surface plasmon polaritons using a phase transition material. *Sci. Rep.* **7**, 43723; doi: 10.1038/srep43723 (2017).

**Publisher's note:** Springer Nature remains neutral with regard to jurisdictional claims in published maps and institutional affiliations.

## Supplementary Material

Supplementary Information

## Figures and Tables

**Figure 1:**
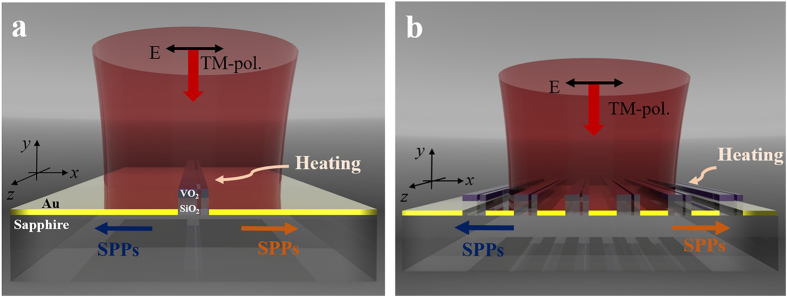
Schematic illustrations of (**a**) an asymmetric VIM nanoantenna and (**b**) a periodic VIM metagrating for the IMT assisted switching of directionally launched SPPs. The left navy arrow and the right orange arrow denote directionally launched SPPs when the VIM nanoantenna is in the insulator and the metal phases, respectively. Light with transverse-magnetic (TM) polarization illuminates normally on the proposed schemes.
