# Supplementary material for: Active directional switching of surface plasmon polaritons using a phase transition material
Source: Sci Rep. 2017 Mar 6;7:43723. doi: 10.1038/srep43723 (PMC5338025; doi:10.1038/srep43723)
Supplement: Supplementary Information [file srep43723-s1.pdf]

Supplementary Information

Active directional switching of surface plasmon polaritons using a phase transition material

Sun-Je Kim[1], Hansik Yun[1], Kyungsoo Park[2], Jongwoo Hong[1], Jeong-Geun Yun[1], Kyookeun Lee[1], Joonsoo Kim[1], Sun Jae Jeong[2], Sang-Eun Mun[1], Jangwoon Sung[1], Yong Wook Lee[2], and Byoungho Lee[1,]*

[1]Inter-University Semiconductor Research Center and School of Electrical and Computer Engineering, Seoul National University, Gwanak-Gu Gwanakro 1, Seoul 08826, Korea

[2]Interdisciplinary Program of Biomedical Mechanical & Electrical Engineering and School of Electrical Engineering, Pukyong National University, Yongso-ro 45, Nam-Gu, Busan 48513, South Korea

*Corresponding Author's E–mail: Byoungho@snu.ac.kr

Part 1. Perturbation effects of an asymmetrically inserted nanoslit in the VIM nanoantenna

Asymmetric insertion of a nanoslit in the VIM nanoantenna yields directional launching of SPPs both in the insulator and metal phases of VO_2. We expected that the insertion would play a role of small perturbation on backside transmissive light scattering leaving portion of the zeroth order transmission, the largest scattering direction, nearly unchanged. As shown in Fig. S1(a) and S1(b), while backside SPPs are directionally launched and switched, the main stream of transmission, the 0th order transmission of light, is not perturbed much.

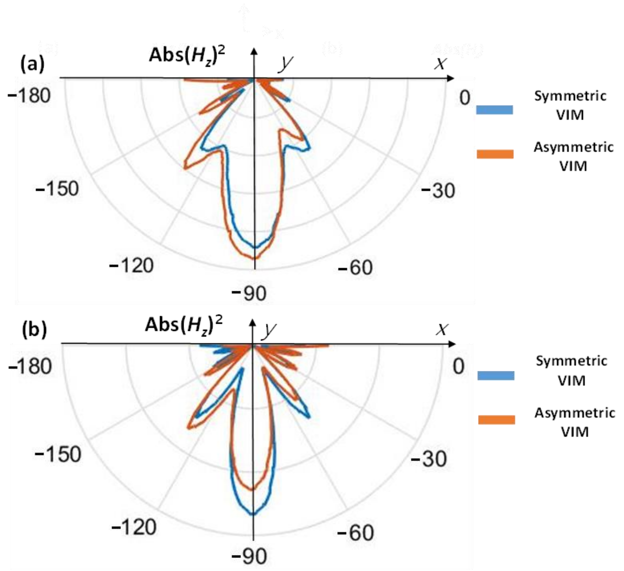

Figure S1. Spatial distributions of squared magnitudes of transverse magnetic fields which are scattered by symmetric and asymmetric VIM nanoantennas in (a) the insulator and (b) metal phases. Field quantities calculated by simulations are measured along the half circle with a 5 µm radius. The symmetric and asymmetric VIM nanoantennas are located at the center of the half circle. For both symmetric and asymmetric VIM nanoantennas, w_{vo2} is fixed to 750 nm. For the asymmetric VIM nanoantenna, w_{slit} and x_{slit} are 150 and 100 nm, respectively.

Part 2. Design of the asymmetric VIM nanoantenna

The directivities of backside surface plasmon polaritons (SPPs) caused by the asymmetry of the VIM nanoantenna are dependent on w_{slit} and x_{slit} (w_{vo2} = 750 nm). As shown in Fig. S2(a) and S2(b), power distinction ratios with different directivities are maximized with different values of w_{slit}, while large x_{slit} is preferred in common. We choose optimal values of w_{slit} and x_{slit} to be 150 nm and 100 nm to achieve similar distinction ratios in the both phases of VO_2.

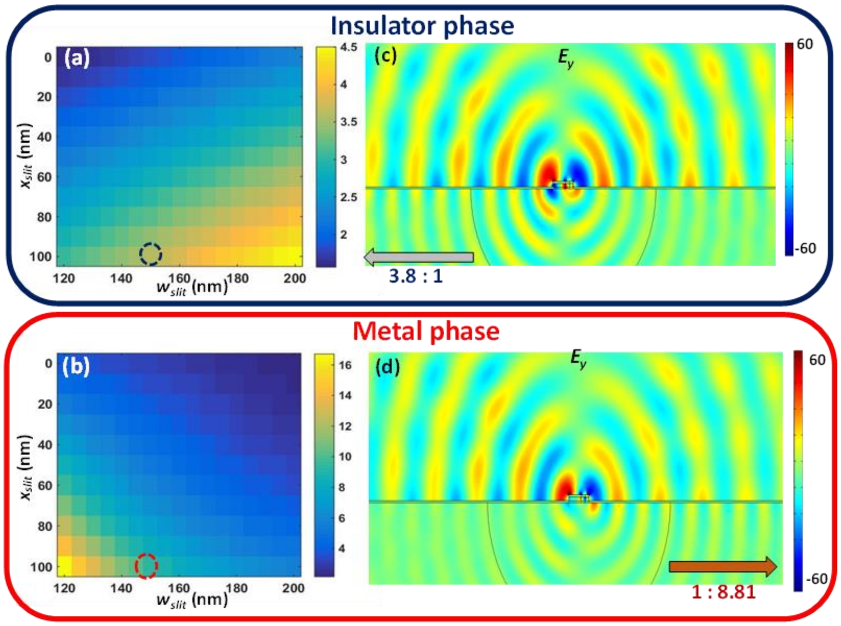

Figure S2. (a) Left-to-right power distinction ratio of backside SPPs in the insulator phase according to variations of w_{slit} and x_{slit} (w_{vo2} = 750 nm). (b) Right-to-left power distinction ratio of backside SPPs in the metal phase according to variations of w_{slit} and x_{slit} (w_{vo2} = 750 nm). E_y-field profiles in (c) the insulator phase and (d) the metal phase respectively, when the VIM antenna of w_{slit} = 150 nm and x_{slit} = 100 nm is illuminated by the tightly focused Gaussian beam with a waist of 5 μm. Left to right power distinction ratio of backside SPPs in the insulator phase, marked as dotted navy circle in (c), is about 3.8. Right to left power distinction ratio of backside SPPs in the metal phase, marked as dotted red circle in (d), is about 8.81.

Part 3. Detailed 5-step fabrication processes

As shown in Fig. S3, fabrication process consists of 5 steps. The first, 150 nm SiO2 layer is deposited on sapphire substrate. Then, 50 nm VO_2 layer is deposited on it. The third step is to make 7 asymmetric VIM nanoantennas for the metagrating and to plane two background regions (45 μm by 15 μm) using an FIB machine. After FIB milling, a 50 nm thick gold film is deposited on it. The last step is another ion beam milling step in order to get rid of gold layers deposited on the VIM metagrating and to make outcoupler gratings on the left and right sides which are 30 μm far from the metagrating. The distances between the device and the grating is chosen to be larger than the beam waist of the tightly focused input laser beam.

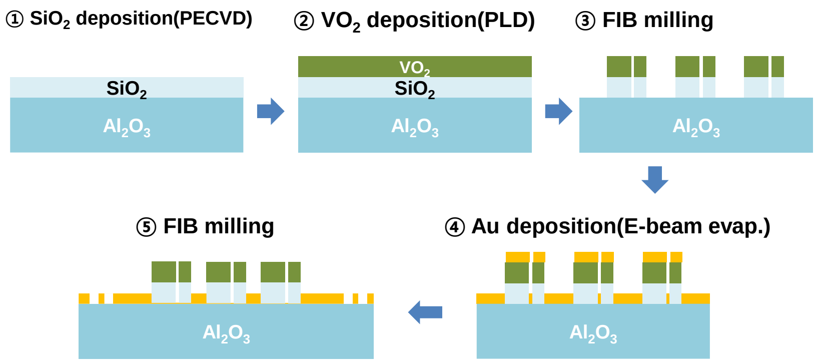

Figure S3. Schematic diagram of 5-step fabrication order for VIM metagrating device. PECVD, PLD, and E-beam evaporation are used for depositions of SiO_2,, VO_2, and Au in the first, the second, and the fourth steps. Ion beam milling is used twice in the third and final steps for shaping the VIM metagrating and air–gold–sapphire regions prepared for SPP propagation.
